# Prolonged Sample Storage Reshapes the m^6^A Methylation Landscape Through RNA Degradation

**DOI:** 10.3390/ijms27083517

**Published:** 2026-04-15

**Authors:** Lingsong Yao, Zhiyu Liu, Ying Wang, Yuwei Yang, Yuqi Sheng, Qinyu Ge, Yunfei Bai

**Affiliations:** State Key Laboratory of Digital Medical Engineering, Southeast University, Nanjing 210096, China; yaolsyy@foxmail.com (L.Y.); 15195986641@163.com (Z.L.); 230218255@seu.edu.cn (Y.W.); yyw.roy.w@gmail.com (Y.Y.); sheng_yuqi@163.com (Y.S.)

**Keywords:** sample storage durations, RNA degradation, N^6^-methyladenosine, RNA integrity

## Abstract

N^6^-methyladenosine (m^6^A) is the most common internal modification in eukaryotic mRNA, essential for post-transcriptional regulation. MeRIP-seq is widely used for m^6^A profiling, but RNA degradation challenges accurate analysis. While the effects of sample handling on RNA are known, the impact of tissue sample storage durations on m^6^A remains unclear. We investigated how sample storage durations (0, 2, 12, 24, and 48 h at room temperature) affect RNA integrity, m^6^A peaks, and transcriptomes in mouse liver. RNA integrity declined with time, reducing m^6^A peak number and reproducibility. Prolonged storage diverged m^6^A profiles, increased unreported peaks, shifted RRACH motifs, and redistributed peaks to intergenic/intronic regions. Integrated data showed opposing changes in m^6^A and expression for some genes, suggesting storage degradation confounds epitranscriptomic interpretation. Sample storage durations are critical for m^6^A accuracy, emphasizing the need for standardized handling in MeRIP-seq studies.

## 1. Introduction 

N^6^-methyladenosine (m^6^A) is the most abundant internal modification of messenger RNA (mRNA) in eukaryotes [1,2,3]. m^6^A modification participates in the regulation of multiple post-transcriptional RNA processes, including splicing, nuclear export, mRNA stability, turnover, and translation [4]. It plays critical roles in cellular differentiation and reprogramming, gametogenesis, embryonic development, stress responses, tumorigenesis, and maintaining cellular integrity by silencing endogenous retroviral-derived RNAs [5,6]. Through these mechanisms, m^6^A plays essential roles in diverse biological processes such as cellular differentiation and lineage commitment, somatic cell reprogramming, gametogenesis, embryonic development, circadian rhythm regulation, stress responses, and immune regulation [7,8,9]. Dysregulation of m^6^A homeostasis has also been implicated in tumor initiation, progression, therapeutic resistance, and maintenance of cancer stem cell properties across multiple cancer types [10,11]. In addition, m^6^A contributes to the maintenance of cellular integrity by suppressing aberrant expression of endogenous retroviral-derived RNAs and repetitive elements, thereby preventing inappropriate innate immune activation [6,12,13].

Currently, methods to detect m^6^A include high-throughput sequencing, colorimetric assays, and liquid chromatography–mass spectrometry (LC-MS) techniques [14,15]. Colorimetric assays and LC-MS permit measurement of the global m^6^A level in mRNA. Meanwhile, LC-MS/MS (tandem mass spectrometry) further allows for simultaneous qualitative and quantitative analysis of bases via molecular ion peaks and fragment ion peaks. However, neither of these approaches provides positional information of m^6^A sites, and they are typically used during the early phases of a project. High-throughput sequencing approaches, especially m^6^A-seq/MeRIP-seq, are currently the most widely used technologies for the study of m^6^A modification [1,16]. MeRIP-seq combines methylated RNA immunoprecipitation (MeDIP), RNA immunoprecipitation (RIP), and RNA-seq technologies to detect RNA methylation at the genome- or transcriptome-wide level with relatively high precision [17]. The principle is that an antibody specific for m^6^A-modified RNA fragments immunoprecipitates RNA carrying m^6^A, and the precipitated RNA fragments are then sequenced by high-throughput methods. Bioinformatic analysis enables a systematic investigation of m^6^A modifications across the transcriptome.

Compared with DNA, RNA is highly unstable and prone to degradation. This presents a challenge for methylation studies of clinical samples [18]. The Agilent 2100 Bioanalyzer is commonly recommended for assessing RNA integrity; samples with an RNA integrity number (RIN) ≥ 8 may be suitable for subsequent RNA methylation library preparation and analysis. However, most clinical samples are collected intra-operatively, and the time window for protective preservation is limited. Sample storage durations (i.e., the time between tissue removal and preservation) may alter mRNA integrity, thereby influencing the accuracy of methylation research results [19].

Previous studies have shown that sample storage durations, storage temperature, and preservation methods can substantially affect RNA integrity and gene expression measurements [20,21,22]. Nevertheless, the potential impact of tissue sample storage durations on RNA methylation profiling, particularly m^6^A distribution and peak reproducibility, has not been systematically investigated. No published studies have specifically examined how different sample storage durations influence m^6^A methylation landscapes. In this context, this study aims to evaluate the effects of distinct sample storage durations (0, 2, 12, 24, and 48 h at room temperature) on RNA integrity and m^6^A profiling outcomes in mouse liver tissue. This work systematically assessed changes in m^6^A peak characteristics and transcriptomic features, seeking to establish a methodological reference for optimizing sample handling and improving the accuracy and reproducibility of MeRIP-seq-based epitranscriptomic studies.

## 2. Results

### 2.1. Effect of Sample Storage Durations on RNA Integrity

**Figure 1:**
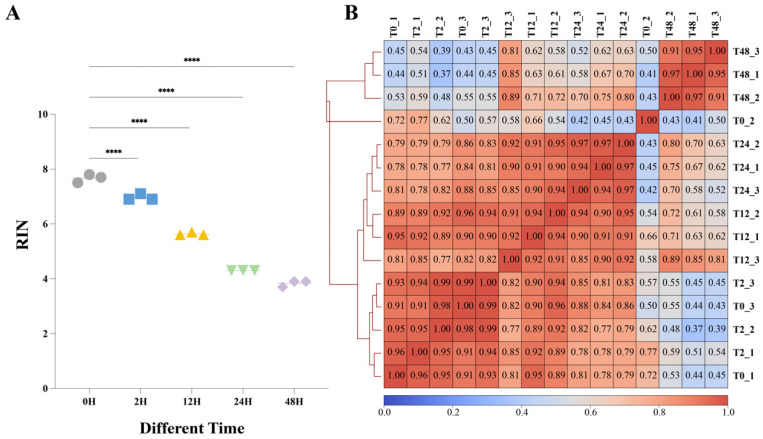
Changes in RNA extracted from samples after different storage durations and the similarity of peaks. (**A**) Changes in RNA extracted from samples after different storage durations. **** indicates a *p*-value < 0.0001. Gray circles represent the RINs for 0H, blue squares represent the RINs for 2H, yellow equilateral triangles represent the RINs for 12H, green inverted triangles represent the RINs for 24H, and purple rhombuses represent the RINs for 48H. (**B**) The similarity of the detected peaks was observed when the samples were placed for different storage durations.

Liver samples were kept at room temperature (25 °C) for 0, 2, 12, 24, and 48 h before RNA extraction to assess the impact of different sample storage durations on RNA integrity in mouse liver tissues. RNA integrity was evaluated using the Agilent 2100 Bioanalyzer by examining the 18S and 28S ribosomal RNA peaks [23,24]. As shown in Figure 1A, liver samples placed for 0 and 2 h exhibited high RNA integrity, whereas samples left for 12, 24, and 48 h showed progressively reduced integrity.

RNA integrity data were statistically analyzed using SPSS (version 26.0). One-way ANOVA was employed to assess the significance of differences between groups. A *p*-value of <0.05 was defined as statistically significant. RIN was significantly decreased compared with the 0 h group at 2 h (*p* < 0.001), 12 h (*p* < 0.001), 24 h (*p* < 0.001), and 48 h (*p* < 0.001). These results indicate that RNA integrity declined progressively with prolonged tissue sample storage durations, consistent with previous studies [25].

Subsequently, RNA from each time point was used for fragmented RIP library construction. After DNA removal and purification, RNA fragments of 100–200 nt were used to prepare input and m^6^A-enriched libraries (approximately 50 µg per library) using Millipore anti-m^6^A antibodies [1,26]. The libraries were then subjected to high-throughput sequencing for downstream data analysis, enabling comparison of m^6^A methylation patterns across the different sample storage durations.

### 2.2. Methylation Features of Peaks Detected at Different Sample Storage Durations

#### 2.2.1. Similarity of Peaks Detected at Different Sample Storage Durations

It is challenging to maintain consistency between biological replicates in chip-seq experiments due to the sensitivity of antibody reactions. We also evaluated sequencing data to ensure high reproducibility across the samples, in addition to standardizing the experimental procedures. For datasets such as chip-seq lacking explicit quantitative results, the standard approach involves partitioning the genome into uniform-length intervals, calculating coverage metrics within each interval, and then assessing sample correlations via comparative analysis of coverage levels across different samples. In this study, we evaluated the consistency of m^6^A profiles across sample storage durations. The pairwise correlations of peak coverage were calculated for samples placed at 0, 2, 12, 24, and 48 h (Figure 1B). Samples processed at 0 and 2 h exhibited very high similarity, with most correlation coefficients being > 0.9. Correlations between the 0 h baseline and samples stored for longer durations decreased progressively. By 12 h, the similarity was substantially reduced, and by 48 h, the correlation with the 0 h profile had dropped below 0.5. These results indicate that prolonged sample storage durations caused a marked divergence from the baseline m^6^A landscape, demonstrating that the sample handling time is a major determinant of reproducibility in MeRIP-seq results.

#### 2.2.2. Number of Detected m^6^A Peaks at Different Sample Storage Durations

**Figure 2:**
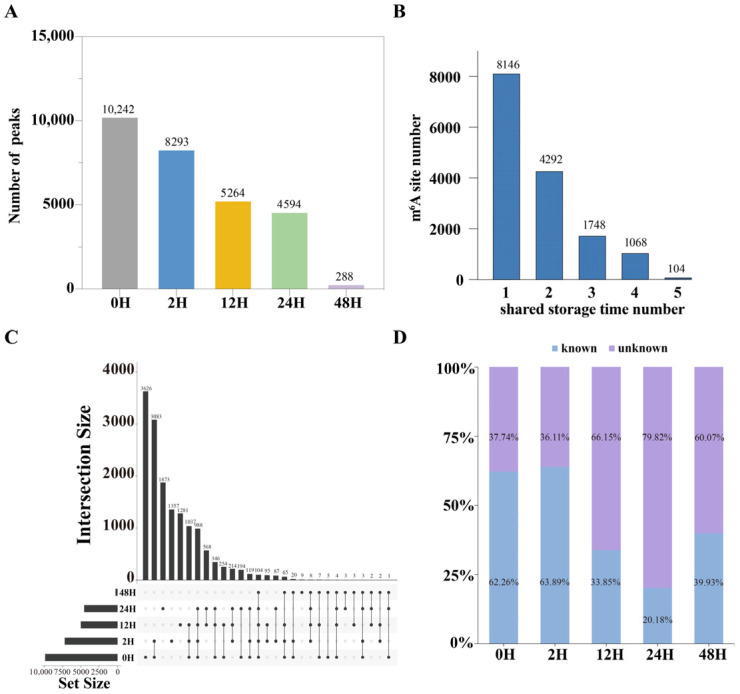
The status of detecting the peak was observed after the samples were placed for different storage durations. (**A**) The number of peaks detected after the samples were placed for different storage durations. (**B**) The distribution of the number of peaks detected at different sample storage durations. (**C**) The samples were placed for different storage durations, and the overlapping state of the detected peaks was observed. (**D**) Comparison of peaks detected after different sample storage durations with RMBase.

m^6^A profiling was performed on liver RNA from mice whose tissues were left at room temperature for 0, 2, 12, 24, and 48 h to evaluate the effect of sample storage durations on m^6^A methylation detection. The results revealed that the number of detected m^6^A peaks decreased progressively with prolonged sample storage durations (Figure 2A). Specifically, 10,242 peaks were identified in samples placed for 0 h, while only 5265 peaks were detected after 12 h, representing nearly a 50% reduction. When the samples were placed for 48 h, merely 288 peaks could be detected. This downward trend closely paralleled the decline observed in the RNA integrity number (RIN) [21].

An intersection analysis was performed to further explore the overlap of detected peaks across the different sample storage durations (Figure 2B). The majority of m^6^A peaks were found to be unique to a single placement time, indicating time-dependent changes in detectable methylation sites. The greatest number of shared peaks (3083) occurred between samples placed for 0 and 2 h, whereas the 48 h samples exhibited very few peaks in common with those from the other four time points (Figure 2C).

#### 2.2.3. Comparison of Peaks Detected at Different Sample Storage Durations with Reported Peaks in RMBase

We compared all detected peaks with previously reported m^6^A sites in the RMBase database (Figure 2D). In samples processed at 0 and 2 h, the majority of detected peaks corresponded to known sites, accounting for more than 60% of all peaks. In contrast, the proportion of unreported peaks increased substantially at later placement times: 66.15%, 79.82%, and 60.07% of peaks detected at 12, 24, and 48 h, respectively, were not present in RMBase. These findings indicate that longer sample storage durations are associated with an increased likelihood of detecting previously unreported peaks, suggesting a growing contribution of degradation-related or artifact-associated signals as sample storage durations increase [27,28].

#### 2.2.4. Motifs Identified from Peaks Detected at Different Sample Storage Durations

**Figure 3:**
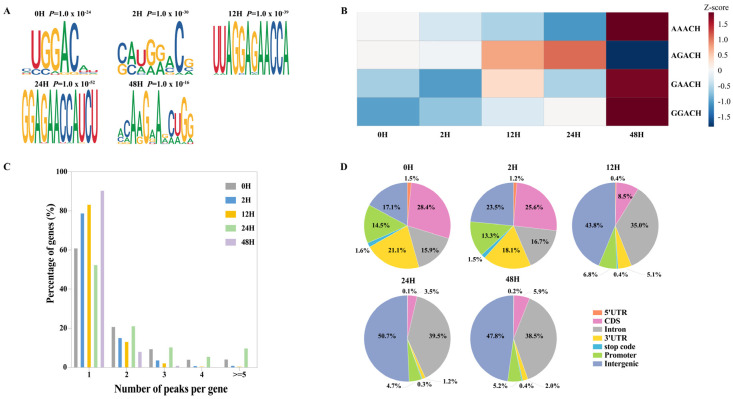
Analysis of peak detection at different sample storage durations. (**A**) The motifs that detected the peak were observed at different time points for the samples. The *p*-value represents the probability of observing a specific motif match within a random sequence. A smaller *p*-value indicates that the motif is less likely to occur by chance, signifying a more statistically significant level of enrichment. (**B**) Heatmap showing the normalized proportions of the four m^6^A motifs in the peaks detected at different sample storage durations. (**C**) The proportion of genes with different numbers of peaks after being placed for different sample storage durations. (**D**) The distribution of the peaks on the gene elements was detected after the samples were placed for different storage durations.

HOMER was used to identify high-confidence sequence motifs within the m^6^A peaks detected from samples placed for different storage durations. The predicted motifs were ranked based on their corresponding *p*-values (Figure 3A). The canonical m^6^A consensus motif RR(m^6^A)CH was consistently detected across all placement groups. For samples placed within 2 h, the predominant motif was GG(m^6^A)CH. As the sample storage durations increased to 24 h, the most enriched motif shifted to GA(m^6^A)CH, and after 48 h, the dominant motif further shifted to AA(m^6^A)CH.

Although the general RRACH consensus was abundant across all groups, the composition of the dominant sub-motifs varied between different sample storage durations. To further examine the dynamics of motif changes, the RRACH motif was subdivided into four sub-motifs: GGACH, AGACH, GAACH, and AAACH. Analysis of these sub-motifs revealed clear temporal preferences in methylation patterns (Figure 3B). The AGACH motif showed a pronounced enrichment after 12 h of placement, but its abundance decreased again after 48 h. In contrast, the motifs AAACH, GAACH, and GGACH were weakly represented at earlier time points but became strongly enriched at 48 h. These findings are consistent with the motif-shift patterns described above, collectively indicating that RNA degradation associated with prolonged sample storage durations may influence the detectable motif landscape of m^6^A-modified peaks.

#### 2.2.5. Distribution Characteristics of Peaks Detected at Different Sample Storage Durations

Genes containing only one m^6^A peak accounted for most methylated genes at all sample storage durations, consistent with previous studies (Figure 3C). With increasing placement time, the number of genes carrying a single peak gradually increased, decreased at 24 h, and rose again at 48 h. In contrast, the number of genes containing two or more peaks showed an overall decline as sample storage durations increased, with a slight rise at 24 h followed by a decrease at 48 h.

The distribution of peaks across gene regions also changed markedly with sample storage durations (Figure 3D). In samples processed at 0 and 2 h, most peaks were enriched in coding sequences (CDSs), followed by intergenic regions and the 3′UTR. However, beginning at 12 h, the distribution pattern shifted: peaks became predominantly enriched in intergenic regions, with intronic regions becoming the second most abundant, while CDS-associated peaks decreased substantially.

This shift is consistent with RNA degradation occurring during prolonged sample storage durations. Coding regions often contain structured elements that may become fragmented earlier during decay, reducing detectable CDS peaks, whereas some intergenic and intronic fragments may remain relatively stable and accumulate in degraded samples. Loss of RNA quality control after cell death may also allow unspliced pre-mRNAs to persist, increasing the proportion of intronic peaks. Additionally, degradation of 3′UTRs by exonucleases likely contributes to the reduced detection of peaks in these regions at later timepoints.

Together, these results indicate that extended sample storage durations reduce overall RNA integrity as well as profoundly alter the genomic distribution of detectable m^6^A peaks, shifting them from typical locations such as CDS and 3′UTRs toward intergenic and intronic regions.

### 2.3. Distribution of Differentially Methylated m^6^A Peaks

Comparing RNA methylation profiles across different experimental conditions can reveal the dynamic nature of post-transcriptional m^6^A regulation in varying sample-handling environments [29,30]. In this section, we examine how different sample storage durations influenced m^6^A detection outcomes to determine how sample processing conditions may alter the landscape of methylation.

#### 2.3.1. Number of Differentially Methylated Peaks

**Figure 4:**
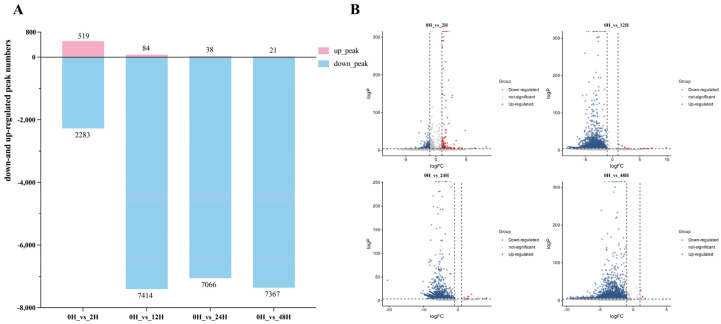
The number of differential peaks detected and volcano plots after different sample storage durations. (**A**) The number of detected differential peaks after different sample storage durations. (**B**) Volcano plot showing the detected differences in peaks after samples were placed for different storage durations.

Pairwise comparisons were performed between samples placed for 0, 2, 12, 24, and 48 h (Figure 4A). Most differential peaks were observed when comparing samples processed immediately (0 h) with those processed after longer sample storage durations. Most of these differential peaks were downregulated relative to the 0 h group.

After 2 h of placement, 519 peaks were significantly upregulated compared with 0 h; however, the number of upregulated peaks progressively declined with longer sample storage durations. In contrast, 2283 peaks were significantly downregulated at 2 h, and this number increased further with extended sample storage durations. After 12 h, the number of downregulated peaks began to decrease, but at 48 h, the number increased again (Figure 4B), reflecting a biphasic pattern likely driven by progressive RNA degradation and altered detectability of methylation sites.

Differential peaks were identified using “log_2_FC ≥ 1 and *p* < 0.05” as the threshold for significantly upregulated methylation sites and “log_2_FC ≤ –1 and *p* < 0.05” for significantly downregulated peaks. Subsequent analyses focused on the characteristics of differential peaks between 0 h and each of the longer sample storage durations (2, 12, 24, and 48 h), as well as the features of peaks shared across all five conditions.

#### 2.3.2. Motifs of Differentially Methylated Peaks

**Figure 5:**
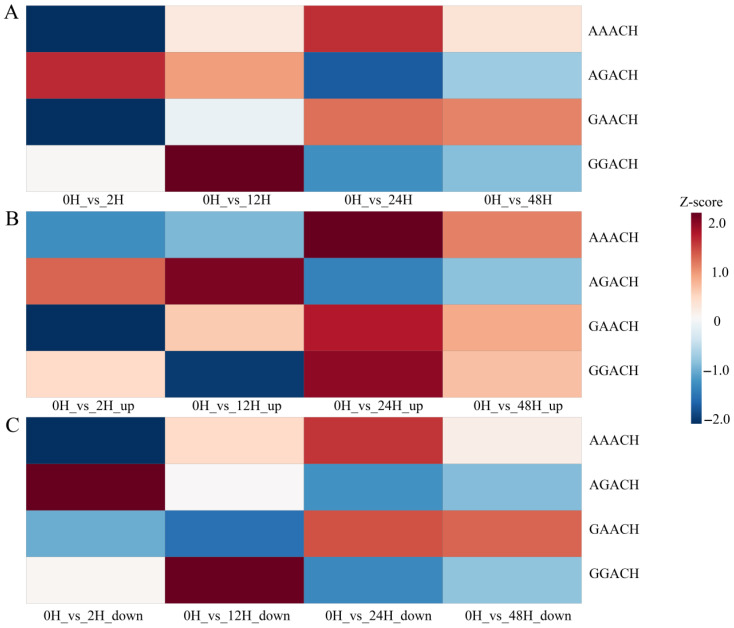
Heatmap showing the normalized proportions of four m^6^A motifs in the detected differential peaks after different sample storage durations. (**A**) Heatmap showing the normalized proportions of four m^6^A motifs in the detected differential peaks after samples were placed for different durations. (**B**) Heatmap showing the normalized proportions of four m^6^A motifs in the differentially upregulated peaks detected at different sample storage durations. (**C**) Heatmap showing the normalized proportions of four m^6^A motifs in the downregulated peaks detected at different sample storage durations.

Motif analysis was performed on the differentially methylated peaks identified between the 0 h group and each of the four extended sample storage durations (Figure 5). All four RRACH submotifs displayed distinct preferences in different comparison groups. The AAACH motif was strongly enriched only in the differential peaks between 0 h and 24 h, as well as between 0 h and 48 h. In contrast, AGACH was predominantly enriched in the differential peaks between 0 h and 2 h, whereas GGACH showed strong enrichment in the comparison between 0 h and 48 h. These findings are consistent with the motif shifts observed in Section 2.2.4, further supporting the transition in dominant RRACH submotifs from GGACH at shorter placement times to AAACH at longer sample storage durations.

### 2.4. Transcriptomic Changes Associated with Different Sample Storage Durations

**Figure 6:**
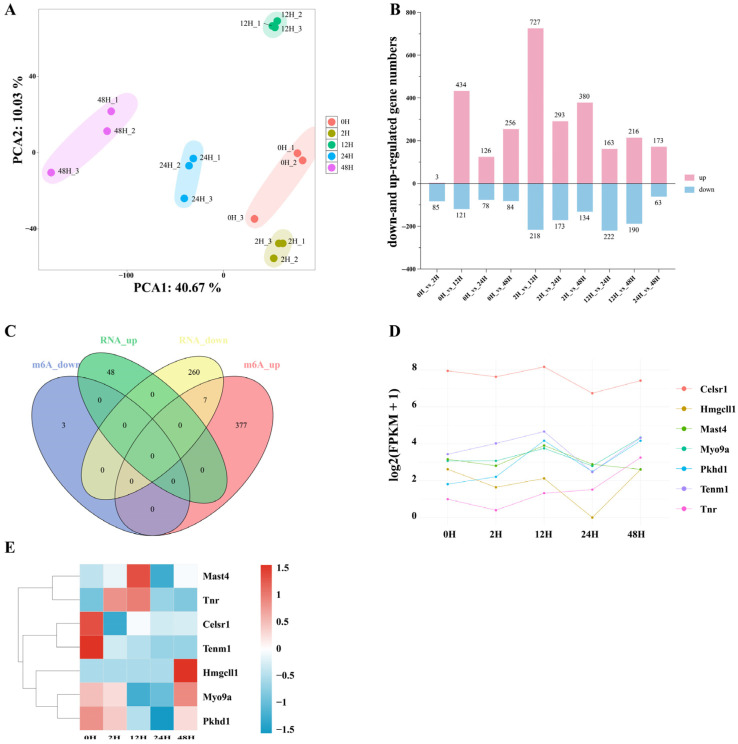
Transcriptomics analysis of samples after different storage durations. (**A**) Principal component analysis diagram of samples placed for different storage durations. (**B**) The number of differentially expressed genes in samples after being placed for different storage durations. (**C**) Venn diagram showing the differences in methylated genes and expressed genes between samples placed for different storage durations. (**D**) The changes in expression of the common genes shared by the differentially methylated genes and the differentially expressed genes in samples with different sample storage durations. (**E**) Heatmap showing the methylation levels of the common genes shared by the differentially methylated genes and the differentially expressed genes at different sample storage durations.

RNA degradation and cell death-associated processes occurring after tissue collection can substantially alter gene expression patterns, potentially confounding biological interpretation, especially in disease-related studies. We performed principal component analysis across all samples to evaluate transcriptional variability introduced by storage durations. The results showed that the major axes of variation were strongly correlated with sample storage durations (Figure 6A), indicating that storage durations are a dominant driver of transcriptome differences.

Differential gene expression analysis was then conducted using |log2FC| ≥ 1 and *p* < 0.05 as thresholds (Figure 6B). After 2 h of placement, the number of differentially expressed genes increased progressively with storage durations, peaking at 12 h. This number decreased after 12 h but rose again after 24 h, reflecting the complex dynamics of RNA degradation and stress-induced transcriptional changes.

### 2.5. Integrated Analysis of Transcriptome and m^6^A Methylation Under Different Sample Storage Durations

In the transcriptome sequencing results, differentially expressed genes (DEGs) were defined as those with |log2FC| ≥ 1 and *p* < 0.05. Similarly, in the MeRIP sequencing data, differentially methylated m^6^A peaks were defined using the same criteria (|log2FC| ≥ 1 and *p* < 0.05), and their corresponding genes were identified. We then selected genes that were shared between the DEGs and differentially methylated genes at 48 h and compared them across different sample storage durations. Correlation analyses were performed to integrate transcriptome and MeRIP-seq data, allowing us to examine the relationship between transcriptional changes and m^6^A methylation alterations. As shown in Figure 6C, seven genes were found to overlap between the shared upregulated methylated genes and shared downregulated expression genes.

**Figure 7:**
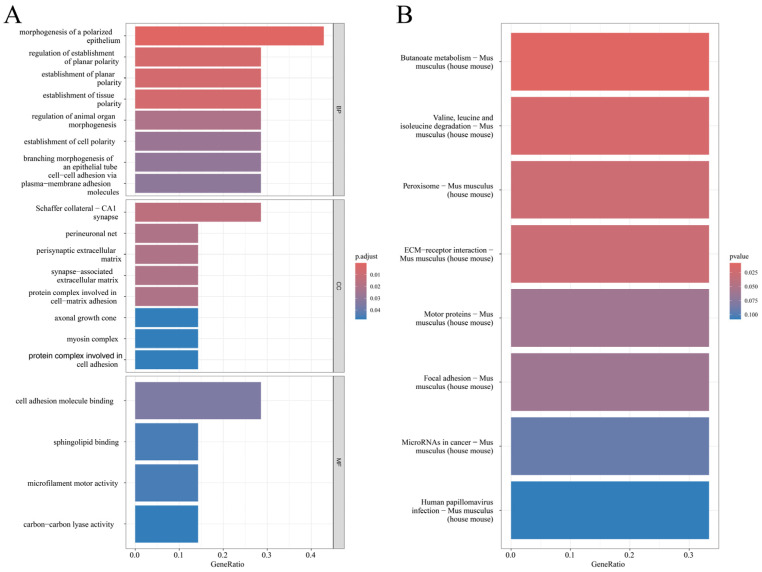
The expression of common genes shared by differentially methylated genes and differentially expressed genes in samples with different storage durations. (**A**) Analysis of GO for differentially methylated genes based on sample storage durations. (**B**) Analysis of KEGG for differentially methylated genes based on sample storage durations.

We assessed the expression levels and m^6^A methylation status of these seven genes at different sample storage durations. As shown in Figure 6D, the expression of Celsr1, Mast4, Hmgcll1, and Tnr initially decreased and then increased, whereas Tenm1, Myo9a, and Pkhd1 initially increased and then decreased. In contrast, the methylation levels of these seven genes showed trends opposite to those observed at the transcriptome level (Figure 6E), suggesting that changes in m^6^A methylation may influence their mRNA stability and steady-state expression levels via post-transcriptional regulatory mechanisms. GO functional enrichment analysis of these genes (Figure 7A) indicated that in the biological process category, they are primarily associated with cell polarity establishment; in the cellular component category, they are mainly localized to synaptic junctions; and in the molecular function category, they are enriched for cell adhesion molecule binding. KEGG pathway analysis further revealed that these genes are mainly involved in metabolic processes, peroxisome function, and ECM–receptor interactions (Figure 7B).

## 3. Discussion

Previous studies have demonstrated that both warm and cold ischemia at room temperature can severely compromise RNA integrity, and several reports have shown that storage durations markedly affect RNA quality and yield [31,32]. While transcriptomic consequences of storage-related degradation have been reported in multiple tissues, the extent to which sample storage durations influence m^6^A methylation has remained unclear. In this study, we systematically examined the impact of different sample storage durations (0, 2, 12, 24, and 48 h) on m^6^A profiles in mouse liver tissue and assessed these changes alongside transcriptomic alterations.

Consistent with earlier observations, RNA integrity decreased progressively with prolonged storage, reflecting ongoing degradation caused by intrinsic RNA structural instability and ubiquitous RNases [33]. The decline in RIN values paralleled the reduction in detectable m^6^A peaks, indicating that loss of RNA integrity likely underlies the diminishing peak count. Comparison of detected peaks with the RMBase database further revealed that extended storage (≥12 h) increased the proportion of newly detected, unreported peaks, suggesting that degradation-induced artifacts become more prominent over time. We hypothesize that prolonged sample storage durations lead to RNA fragmentation, which alters the enrichment efficiency of MeRIP antibodies at specific sites, resulting in their misidentification as unreported peaks.

Motif analysis across all detected peaks revealed the canonical RR(m^6^A)CH sequence [16]. However, the dominant sub-motifs shifted with storage durations: GG(m^6^A)CH within 2 h, transitioning to GA(m^6^A)CH at 24 h, and eventually to AA(m^6^A)CH by 48 h. This progressive substitution of G with A suggests that degradation may remodel the sequence context in which m^6^A is detectable. Non-random fragmentation at room temperature may lead to the loss of RNA fragments originally enriched with GG sequences due to their short length during enrichment processes. Concurrently, if GA/AA sites are situated within more stable sequence contexts, their survival rate in degraded samples would be significantly higher than that of GG sites. The peak distribution also changed markedly over time. At 0–2 h, most peaks were located in CDS regions, followed by intergenic elements and 3′UTRs [34]. Beyond 12 h, peaks became increasingly enriched in intergenic and intronic regions, with a steady reduction in CDS-associated signals, an observation similar to transcriptome studies using degraded RNA [35]. Given that intronic reads largely originate from unspliced pre-mRNA, these findings imply that deteriorating cellular conditions during extended storage may impede splicing fidelity, causing accumulation of immature transcripts and altering detectable m^6^A patterns [36]. Such changes, especially after 2 h, could obscure genuine biological methylation signals [37].

Analysis of differentially methylated peaks further demonstrated consistent enrichment of the RRACH motif and localization predominantly within CDS and 3′UTRs. Functional enrichment revealed that before 12 h, up-methylated genes were strongly associated with stimulus response pathways, whereas localization and general cellular processes were uniquely enriched during early time points. By 48 h, up-methylated genes were mainly associated with positive regulation of biological processes, while genes involved in cell-to-cell interactions and development appeared only after 24 h. These patterns may reflect transient cellular activity during early storage when metabolic and response pathways remain partially functional, followed by decline as degradation progresses. Down-methylated genes were consistently enriched in cell process, metabolic, developmental, and regulatory pathways across time points, with enrichment intensity increasing with prolonged storage, further supporting the notion that degradation-driven dysfunction intensifies over time.

We evaluated how sample storage durations influenced the transcriptome to further dissect the relationship between m^6^A regulation and transcriptional output. PCA revealed that storage duration was a major driver of global transcriptional variation. DEG analyses indicated dynamic changes: increasing DEG numbers with prolonged sample storage durations up to 12 h, a temporary reduction at 12 h, and a renewed increase by 24 h. Integrating methylation and transcriptional data revealed seven genes whose methylation increased while their expression decreased across storage durations. Given the opposing trends between methylation and expression, these genes may represent cases in which altered m^6^A deposition modulates transcript stability or translation, ultimately shaping expression levels during storage-induced degradation [38].

## 4. Materials and Methods

### 4.1. Animals and Sample Collection

C57Bl/6J mouse (8 weeks old) was anesthetized with tribromoethanol (500 mg/kg) (Sigma, Saint Louis, MO, USA) and then was killed by cervical dislocation. The liver was dissected and then washed with 0.9% pre-cooled saline. We extracted RNA from all 5 groups (with 3 replicates for each group), and the samples were stored at room temperature (RT) for 0, 2, 12, 24, and 48 h before RNA extraction. This study was reviewed and approved by the Ethics Committee of Zhongda Hospital, Southeast University.

### 4.2. MeRIP Sequencing and RNA Sequencing

Total RNA was isolated and purified using TRIzol™ Reagent (Invitrogen™, cat. no15596018, Waltham, MA, USA) following the procedure of the ‘‘Refined RIP-seq’’, with several modifications [26,39]. An amount of 50 μg of total RNA was fragmented into 200-nucleotide-long fragments using a magnesium RNA fragmentation buffer (Invitrogen™, cat. no AM8740, Waltham, MA, USA). The fragmentation was stopped by adding an RNA fragmentation stop solution, followed by ethanol precipitation. An amount of 10 ng of fragmented total RNA was used as input, and the remaining RNA was used for m^6^A-seq. Briefly, RNA was added to anti-N6-methyladenosine (m^6^A) antibodies (Millipore, cat. no ABE572, Burlington, MA, USA), protein A-magnetic beads (Invitrogen™, cat. no 10002D), and protein G-magnetic beads (Invitrogen™, cat. no 10004D), which were then mixed and incubated overnight. The beads–antibody–RNA mixture was washed and eluted from the beads with 6.7 mM N6-methyladenosine (Sigma-Aldrich, cat. no M2780, St. Louis, MO, USA). RNA was extracted using an RNeasy Mini Kit (QIAGEN, cat. no 74106, Hilden, Germany) [40]. The fragmented total RNA (input) and immunoprecipitated RNA (IP) underwent library construction using a SMARTer Stranded Total RNA-Seq Kit v2—Pico Input Mammalian (Takara-Clontech, cat. no 634413, San Jose, CA, USA), according to the manufacturer’s protocol [41]. RNase inhibition treatment was implemented during the experiment. AN Agilent 2100 Analyzer was used to perform a quality inspection on the library and detect whether the library’s size distribution was consistent with the theoretical size. The NovaSeq high-throughput sequencing platform and PE150 sequencing mode were used [42].

### 4.3. Reads Pre-Processing and Alignment

The strand orientation of the original RNA was preserved during the process of library construction, and reads 2 yielded sequences sense to the original RNA. Thus, only reads 2 was used for m^6^A signal identification in our study. The raw sequencing reads were first subjected to Trim_galore (version 0.6.10) for quality control and trimming adaptors. The quality threshold was set to 20, and the minimum length required for reads after trimming was 30 nt. All reads that mapped to mouse rRNA with TopHat2 (version 2.0.13) were removed. The processed reads were mapped to the genome (mm10, UCSC Genome Browser) using HISAT2 (version 2.1.0) with default parameters and separated by strand with in-house scripts.

### 4.4. Identification of Putative m^6^A Sites

For the genome-based peak caller MACS2 (version 2.1.1), the effective genome size was set to 1.87 × 10^9^ for the mouse genome, under the option of -nomodel and a *p*-value cutoff of 0.01. The read number in all input bam files was normalized to the same. Peaks were annotated with annotatePeaks.pl (Homer version 4.8), and the peak reads’ coverage was shown with IGV (version 2.4.15).

### 4.5. Analysis of RNA-Seq Data

Paired-end, adaptor-clean reads were mapped to the human and mouse genomes (hg19 and mm10, UCSC Genome Browser) using TopHat2 (version 2.0.13) with default parameters. The expression of transcripts was quantified as FPKM with Cufflinks (version 2.2.1).

### 4.6. Motif Discovery and GO Enrichment Analysis

For the analysis of sequence consensus, the top 1000 peaks were chosen for de novo motif analysis with Homer (version 4.8), with 100 nt long peak-summit-centered sense sequences as input. Gene Ontology (GO) enrichment analyses were performed using the DAVID web-based tool (version 6.8).

## Data Availability

The data that support the findings of this study are openly available in NCBI at https://www.ncbi.nlm.nih.gov/sra/PRJNA1449554 (accessed on 7 April 2026).
